# Effect of living area and sports club participation on physical fitness in children: a 4 year longitudinal study

**DOI:** 10.1186/1471-2458-14-499

**Published:** 2014-05-23

**Authors:** Kathleen Golle, Urs Granacher, Martin Hoffmann, Ditmar Wick, Thomas Muehlbauer

**Affiliations:** 1 Division of Training and Movement Sciences, University of Potsdam, Am Neuen Palais 10, House 12, D-14469, Potsdam, Germany; 2 University of Applied Science in Sport and Management, Potsdam, Germany

**Keywords:** Motor performance, Youth, Primary school, Maturation

## Abstract

**Background:**

Cross-sectional studies detected associations between physical fitness, living area, and sports participation in children. Yet, their scientific value is limited because the identification of cause-and-effect relationships is not possible. In a longitudinal approach, we examined the effects of living area and sports club participation on physical fitness development in primary school children from classes 3 to 6.

**Methods:**

One-hundred and seventy-two children (age: 9–12 years; sex: 69 girls, 103 boys) were tested for their physical fitness (i.e., endurance [9-min run], speed [50-m sprint], lower- [triple hop] and upper-extremity muscle strength [1-kg ball push], flexibility [stand-and-reach], and coordination [star coordination run]). Living area (i.e., urban or rural) and sports club participation were assessed using parent questionnaire.

**Results:**

Over the 4 year study period, urban compared to rural children showed significantly better performance development for upper- (*p* = 0.009, *ES* = 0.16) and lower-extremity strength (*p* < 0.001, *ES* = 0.22). Further, significantly better performance development were found for endurance (*p* = 0.08, *ES* = 0.19) and lower-extremity strength (*p* = 0.024, *ES* = 0.23) for children continuously participating in sports clubs compared to their non-participating peers.

**Conclusions:**

Our findings suggest that sport club programs with appealing arrangements appear to represent a good means to promote physical fitness in children living in rural areas.

## Background

Physical fitness is an important health determinant that is related to several physiological functions. Recent studies regarding the health burden of chronic diseases revealed significant inverse associations between physical fitness and various cardiovascular risk factors (e.g., blood pressure, insulin resistance, cholesterol/lipids, overweight/adiposity) in children and adolescents [1-3]. Further, there is evidence that physical fitness and its health-related outcomes track from childhood over adolescence into adulthood [4,5]. In fact, findings from longitudinal studies indicate that higher levels of physical fitness during childhood and adolescence are associated with a healthier cardiovascular profile in adulthood (for a review see [6]).

Several non-modifiable (i.e., genetics) [7] and modifiable factors influence physical fitness. Modifiable factors can be categorized in behavioral (e.g., physical activity, media use) and environmental (e.g., socioeconomic status, living area) subcategories and are thus of particular interest from a health-promoting perspective [8-10]. More specifically, the subcategory living area appears to play an important role in moderating physical fitness. Most of the literature on the association between living area and physical fitness observed better performances for children living in rural compared to urban areas [11-13]. For example, Dollmann et al. [13] showed significantly better results for a measure of endurance (1-mile run) in rural compared to urban children aged 10–11 years. Further, Karkera et al. [12] reported significantly better performances for rural than for urban school children (9–13 years) in tests for endurance (20-m shuttle run) and flexibility (sit-and-reach). Lastly, Chillon et al. [14] observed heterogeneous results in youth (7–13 years) with students from rural areas showing better performances in measures of aerobic capacity (20-m shuttle run), muscle endurance (bent-arm hang), and strength (handgrip force). On the other hand, students from urban areas showed better performances in measures of speed (5 × 10-m shuttle run), flexibility (sit-and-reach), and muscle endurance (sit-ups). Even though the reported studies provide further insight in the association between living area and physical fitness, their added scientific value is limited because findings from cross-sectional studies do not allow the identification of cause-and-effect relationships.

Besides the subcategory living area, sports participation represents another factor that is related to physical fitness [10,15,16]. For example, Ara et al. [15] reported significantly better scores for lower-extremity muscle strength (squat jump), endurance (20-m shuttle run), and speed (sprint tests over 30 and 300 m) in boys (8–11 years) participating in a sport club as compared to their non-participating classmates. In support of this finding, Drenowatz et al. [16] observed significantly better results in sports club participating children (7 ± 1 years) for endurance (6-min run), upper-extremity muscle strength (medicine ball throw), coordination (throw-and-turn), and balance (one-leg balancing) compared to their non-participating peers. However, the above mentioned studies are limited because they applied a cross-sectional and not a longitudinal design. Thus, longitudinal data is needed to provide conclusive evidence whether sports participation and/or living area really represent determinants of physical fitness development in children.

Therefore, this study used a longitudinal approach and we aimed at investigating differences in physical fitness levels over time (i.e., classes 3 to 6) between children living in urban as compared to rural areas and between sports club participating children and their non-participating peers. Based on the literature [11-13], it is hypothesized that physical fitness level and its development is better in children living in rural compared to urban areas. In addition, it is expected that children continuously participating in sports clubs show better physical fitness levels as well as larger fitness development rates than their non-participating peers.

## Methods

### Sample and study design

A longitudinal study was conducted to test changes in physical fitness in the same children over time (i.e., from class 3 to 6). This time frame was chosen because the involved sports clubs searched for physically talented children. The participating children attended public primary schools (*N* = 24) that were randomly selected from urban (i.e., cities > 10,000 inhabitants) and rural (i.e., cities/villages ≤ 10,000 inhabitants) areas of the federal state Brandenburg (Germany). This classification was made according to the federal statistics report [17]. Over the 4-year study, the annual testing period lasted 4 weeks (i.e., from March to April) each year. The study was approved by the Ministry of Education, Youth and Sport of the federal state Brandenburg. Parents or legal representatives of each child provided written informed consent. The study was conducted according to the declaration of Helsinki. Of the initially recruited 341 children, informed consent and valid data were obtained from 172 children (69 girls, 103 boys) over the 4-year period. This data set was used for further analyses.

### Anthropometry

Body height was measured without shoes to the nearest 0.5 cm with a wall-mounted stadiometer (Seca, Basel, Switzerland). Body mass was determined in light clothing and without shoes to the nearest 100 g with an electronic scale (Bodymaster vision BM-210, Rowenta, France). Body mass index (BMI) was calculated using body mass divided by height squared (kg/m^2^).

### Physical fitness testing

Physical fitness was determined with 6 different tests from motor fitness test batteries of Bös [18,19] and Stark [20] in the same children starting in class 3 and repeated in classes 4, 5, and 6. The tests included the following items: 50-m sprint, 1-kg ball push, triple hop, stand-and-reach, star coordination run, and 9-min run. Test-retest reliability of these tests ranged from *r* = 0.70 to 0.94 [18,19,21]. A description of all test items is provided in Table 1. All tests were performed in the respective school gyms following standardized test protocols (e.g., test instructions).

**Table 1:** **Description of all physical fitness test items **[18-20]**and the respective motor ability (in parentheses)**

**Fitness test item**	**Fitness test description**	**Score**
star coordination run (agility)	- minimal time needed to run from the center to the edge and back of a star with 4 spikes	s
9-min run (endurance)	- maximal distance achieved during 9 minutes	m
stand-and-reach (flexibility)	- maximal reach while standing with extended knees	cm
50-m sprint (speed)	- minimal time needed on a 50-m run	s
Triple hop (lower-extremity strength)	- maximal horizontal jump distance	m
1-kg ball push (upper-extremity strength)	- maximal pushing distance	m

### Questionnaire

Parents filled out a questionnaire that was sent home. Questions contained information regarding the subcategories living area (rural or urban) and sports club participation (Yes or No option). Only children that did not change their residential status (*N* = 172; urban: *n* = 89, rural: *n* = 83) over the 4-year study period were included for further analyses. Sixty-six out of those 172 children either continuously participated (*n* = 49) or did not participate (*n* = 17) in a sports club at all. The remaining one-hundred six children changed their status of sports club participation from ‘Yes’ to ‘No’ or vice versa over the study period and were therefore not included in our analysis.

### Statistical analyses

Data are presented as group mean values and standard deviations. Physical fitness parameters were analyzed in separate sex-adjusted analyses of variance (ANOVA) with repeated measures on class. Post-hoc tests with the Bonferroni-adjusted α were conducted to identify the comparisons that were statistically significant. The classification of effect size (*ES*) was determined by calculating partial $\eta_{p}^{2}\text{.}$ According to Cohen [22] *ES* can be classified as small (0.00 ≤ *ES* ≤ 0.24), medium (0.25 ≤ *ES* ≤ 0.39), and large (*ES* ≥ 0.40). In addition, odds ratios (OR) and 95% confidence interval (CI) were calculated using the chi-square test to determine associations between living area and sports club participation. The significance level was set at *p* < 0.05. All analyses were performed using Statistical Package for the Social Sciences (SPSS) version 22.0.

## Results

Anthropometric and physical fitness test data of the study sample sorted by class and sex are presented in Table 2. Boys were significantly taller than girls in classes 3 and 4 (both *p* < 0.05). Eighty-nine children lived in an urban (boys: 51%, girls: 52%) and 83 children in a rural (boys: 49% girls: 48%) area. Over the 4-year experimental period, 49 children (boys: 79%, girls: 67%) continuously participated in a sports club at least once a week while 17 children (boys: 21% girls: 33%) did not participate in a sports club during the whole study period. Thirty-two urban (36%) and 17 rural (20%) children continuously participated in a sports club. The correlative analysis regarding living area and sports club participation revealed that living in rural areas is more likely associated with not participating in a sports club (*n* = 66, OR = 2.7, 95% CI = 0.87-8.33) compared to living in urban areas. The number of practiced sports significantly differed (*p* < 0.001) between urban (i.e., 27 different sports) and rural (i.e., 13 different sports) children.

**Table 2:** Description of the study sample by class and sex

	**3**^**rd **^**class (*N*** **= 172)**		**4**^**th **^**class (*N*** **= 172)**		**5**^**th **^**class (*N*** **= 172)**		**6**^**th **^**class (*N*** **= 172)**	
	**Boys (*n*** **= 103)**	**Girls (*n*** **= 69)**	**Boys (*n*** **= 103)**	**Girls (*n*** **= 69)**	**Boys (*n*** **= 103)**	**Girls (*n*** **= 69)**	**Boys (*n*** **= 103)**	**Girls (*n*** **= 69)**
*Characteristic*								
Age (yr)	9.4 (0.4)*	9.2 (0.4)	10.3 (0.4)*	10.2 (0.4)	11.4 (0.5)*	11.2 (0.3)	12.4 (0.4)*	12.2 (0.4)
Height (cm)	139.8 (5.9)*	136.9 (6.4)	143.6 (6.2)*	141.6 (6.9)	150.0 (6.8)	148.8 (7.7)	156.2 (7.7)	154.9 (7.6)
Mass (kg)	32.0 (5.0)	31.6 (5.9)	35.9 (6.0)	35.9 (7.8)	40.6 (7.4)	40.7 (10.1)	45.7 (8.6)	45.8 (11.2)
BMI (kg/m^2^)	16.4 (2.1)	16.8 (2.4)	17.4 (2.4)	17.8 (3.0)	18.0 (2.5)	18.2 (3.4)	18.6 (2.6)	19.0 (3.7)
*Fitness test*								
50-m sprint (s)	9.6 (0.8)	9.9 (0.8)	9.2 (0.8)*	9.5 (0.9)	8.9 (0.8)	9.1 (0.8)	8.7 (0.8)	8.9 (0.8)
1-kg ball push (m)	7.82 (1.4)*	6.33 (1.4)	9.09 (1.6)*	7.61 (1.3)	10.76 (2.0)*	8.93 (1.9)	11.78 (2.3)*	9.8 (2.2)
Triple hop (m)	7.76 (1.2)*	7.30 (1.1)	8.51 (1.2)*	8.04 (1.2)	9.29 (1.3)*	8.81 (1.2)	9.97 (1.4)*	9.5 (1.4)
SaR (cm)	96.5 (7.8)*	100.7 (7.2)	96.6 (8.1)*	100.6 (7.2)	95.3 (8.5)*	101.6 (7.4)	96.0 (8.3)*	103.9 (7.8)
Star run (s)	22.9 (3.3)	23.6 (2.3)	20.8 (2.0)*	21.8 (1.9)	19.5 (2.0)*	20.4 (1.8)	18.6 (1.8)*	19.3 (1.7)
9-min run (m)	1499.5 (193.1)*	1343.4 (168.3)	1587.9 (255.2)*	1417.1 (213.9)	1629.2 (235.9)*	1455.8 (177.9)	1610.4 (254.7)*	1463.3 (229.3)

*Notes.* Values are means (±SD). *indicates that boys are significantly different from girls (*p* < 0.05); BMI = Body Mass Index; SaR = stand-and-reach test; star run = star coordination run

### Effect of living area on physical fitness development

**Figure 1:**
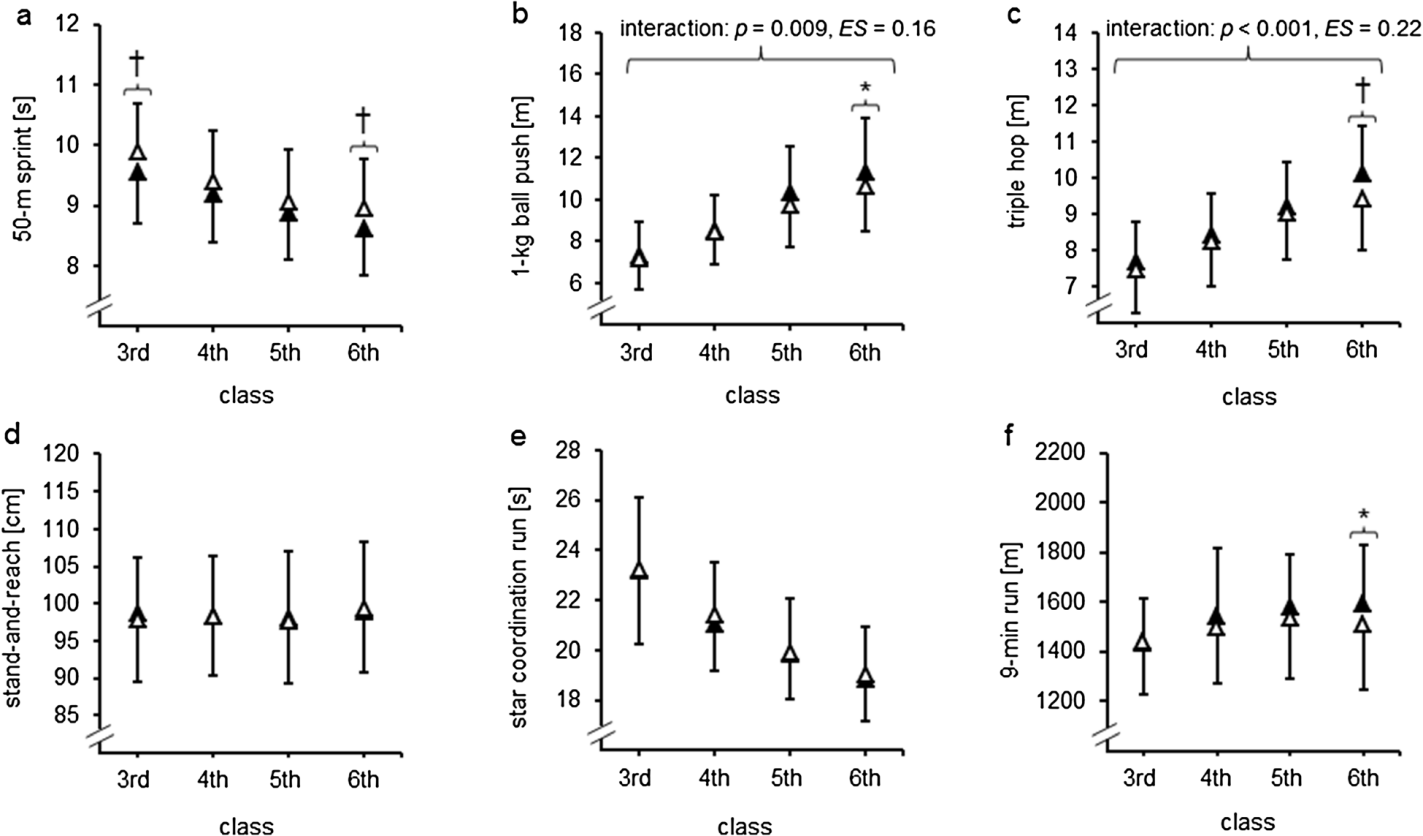
**Development of physical fitness in 172 primary school children (boys: *n*** **= 103, girls: *n*** **= 69) according to class and living area (urban: *n*** **= 89, rural: *n*** **= 83 girls): (a) 50-m sprint, (b) 1-kg ball push, (c) triple hop, (d) stand-and-reach, (e) star coordination run, and (f) 9-min run. ***Notes.* * (*p* < 0.05) and † (*p* < 0.01) indicate that performance was significantly better in urban than in rural children; filled triangles indicate urban and unfilled triangles indicate rural children; for the 50-m sprint and the star coordination run, lower scores indicate better performance; for the 1-kg ball push, the triple hop, the stand-and-reach, and the 9-min run, higher scores indicate better performance.

Statistically significant interaction effects of class x living area were detected for the 1-kg ball push (*F*_[3, 510]_ = 4.2, *p* = 0.009, $\eta_{p}^{2}=0.024$, *ES* = 0.16) and for the triple hop test (*F*_[3, 510]_ = 8.1, *p* < 0.001, $\eta_{p}^{2}=0.046$, *ES* = 0.22) (Table 3). Further, significant main effects of class were found for all physical fitness tests (all *p* ≤ 0.020, *ES* = 0.14-0.62). Additionally, the main effect of living area was significant for the 50-m sprint test (*F*_[1, 170]_ = 5.8, *p* = 0.017, $\eta_{p}^{2}=0.033$, *ES* = 0.18). In sixth graders, post-hoc analyses indicated significantly better performances in 4 out of 6 physical fitness tests (i.e., 50-m sprint, 1-kg ball push, triple hop, 9-min run) in favor of children living in urban compared to rural areas (Figure 1 a-f).

**Table 3:** Results of the ANOVA (adjusted for sex) with repeated measures on class by living area

**Main/interaction effect**	***F*-value**	***df***	***p*-value**	**Partial *η***^**2**^	***ES***
50-m sprint class	17.8	3	0.000	0.095	0.32
50-m sprint area	5.8	1	0.017	0.033	0.18
50-m sprint class x area	1.9	3	0.131	0.011	0.11
1-kg ball push class	65.6	3	0.000	0.280	0.62
1-kg ball push area	2.8	1	0.099	0.016	0.13
1-kg ball push class x area	4.2	3	0.009	0.024	0.16
Triple hop class	52.5	3	0.000	0.237	0.56
Triple hop area	3.4	1	0.066	0.020	0.14
Triple hop class x area	8.1	3	0.000	0.046	0.22
SaR class	6.8	3	0.000	0.039	0.20
SaR area	0.0	1	0.855	0.000	0.00
SaR class x area	0.9	3	0.429	0.005	0.07
Star run class	35.2	3	0.000	0.172	0.46
Star run area	0.4	1	0.534	0.002	0.04
Star run class x area	0.5	3	0.654	0.003	0.05
9-min run class	3.4	3	0.020	0.019	0.14
9-min run area	2.4	1	0.127	0.014	0.12
9-min run class x area	2.4	3	0.074	0.014	0.12

*Notes.* study sample (103 boys, 69 girls); SaR = stand-and-reach test; star run = star coordination run; area = living area; *df* = degrees of freedom; *ES* = effect size.

### Effect of sports club participation on physical fitness development

**Figure 2:**
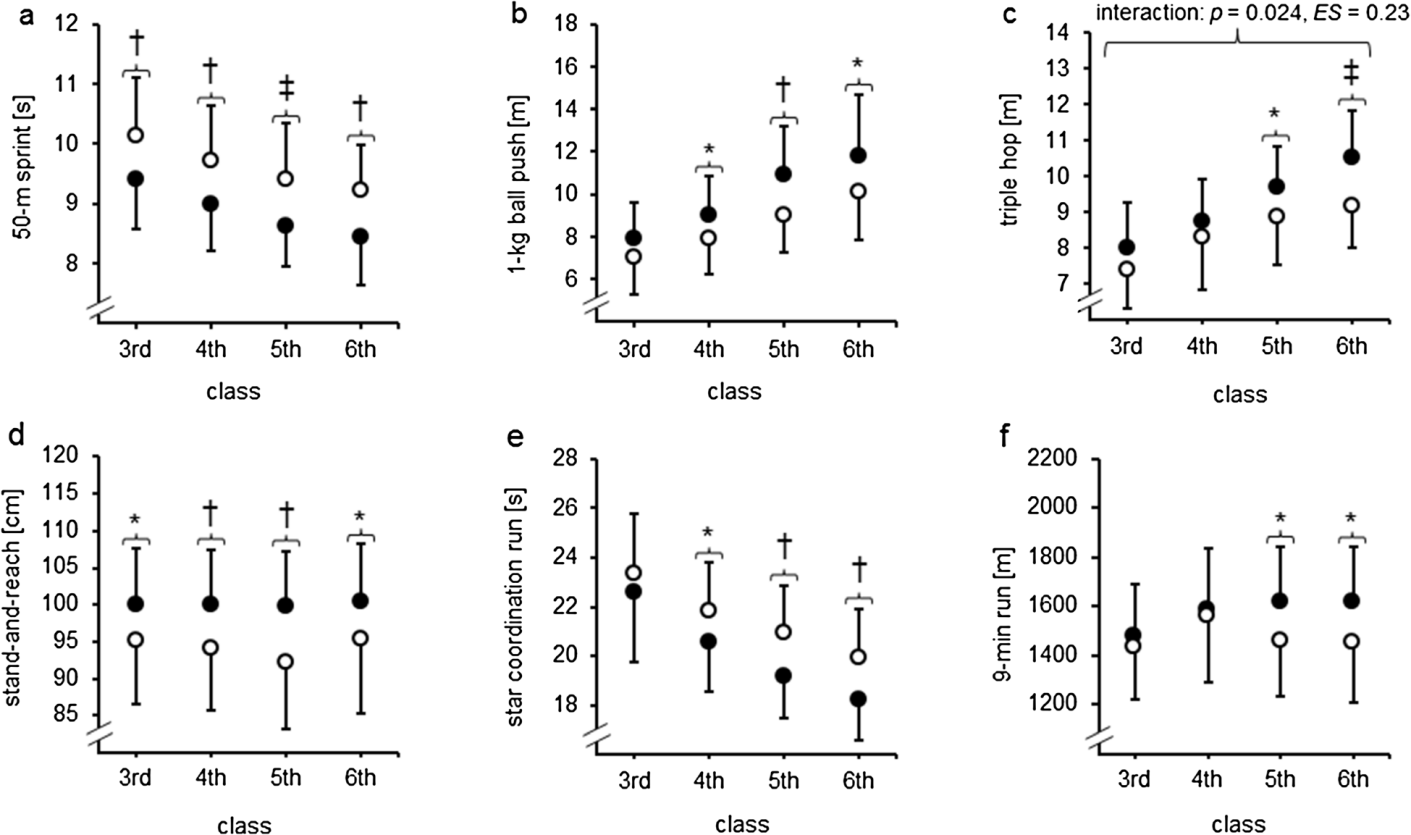
**Development of physical fitness in 66 primary school children (boys: *n*** **= 42, girls: *n*** **= 24) according to class and sports club participation (Yes: *n*** **= 49, No: *n*** **= 17): (a) 50-m sprint, (b) 1-kg ball push, (c) triple hop, (d) stand-and-reach, (e) star coordination run, and (f) 9-min run. ***Notes.* *(*p* < 0.05), †(*p* < 0.01), and ‡(*p* < 0.001) indicate that performance was significantly better in children with than without continuous sports club participation; filled circles indicate children with and unfilled circles indicate children without continuous sports club participation; for the 50-m sprint and the star coordination run, lower scores indicate better performance; for the 1-kg ball push, the triple hop, the stand-and-reach, and the 9-min run, higher scores indicate better performance.

Due to the fact that living in rural areas is more likely associated with not participating in a sports club, the factor living area was included as a covariate in our statistical model. As a result, statistically significant interaction effects of class x sports club participation were detected for the triple hop test (*F*_[3, 192]_ = 3.2, *p* = 0.024, $\eta_{p}^{2}=0.049$, *ES* = 0.23) and for the 9-min run test (*F*_[3, 192]_ = 2.3, *p* = 0.083, $\eta_{p}^{2}=0.035$, *ES* = 0.19) (Table 4). Further, significant main effects of class were observed in all (all *p* ≤ 0.023, *ES* = 0.24-0.30) but 2 physical fitness test (i.e., stand-and-reach, 9-min run). Additionally, the main effect of sports club participation turned out to be significant (all *p* ≤ 0.030, *ES* = 0.28-0.44) in all but 1 physical fitness tests (i.e., 9-min run). Post-hoc analyses indicated significantly better performances in all physical fitness tests for children participating in sport clubs compared to those who did not. This is particularly prevalent for fifth- and sixth-graders (Figure 2 a-f).

**Table 4:** Results of the ANOVA (adjusted for sex and living area) with repeated measures on class by sports club participation

**Main/interaction effect**	***F*-value**	***df***	***p*-value**	**Partial *η***^**2**^	***ES***
50-m sprint class	3.5	3	0.023	0.053	0.24
50-m sprint participation	10.8	1	0.002	0.148	0.42
50-m sprint class x participation	0.1	3	0.915	0.002	0.04
1-kg ball push class	4.3	3	0.011	0.064	0.26
1-kg ball push participation	4.9	1	0.030	0.074	0.28
1-kg ball push class x participation	1.1	3	0.357	0.017	0.13
Triple hop class	5.5	3	0.001	0.081	0.30
Triple hop participation	5.2	1	0.026	0.078	0.29
Triple hop class x participation	3.2	3	0.024	0.049	0.23
SaR class	1.6	3	0.199	0.025	0.16
SaR participation	11.9	1	0.001	0.162	0.44
SaR class x participation	1.7	3	0.167	0.027	0.17
Star run class	3.8	3	0.021	0.057	0.25
Star run participation	6.8	1	0.012	0.098	0.33
Star run class x participation	1.2	3	0.322	0.018	0.14
9-min run class	0.2	3	0.924	0.003	0.06
9-min run participation	1.9	1	0.173	0.030	0.18
9-min run class x participation	2.3	3	0.083	0.035	0.19

*Notes.* study sample (103 boys, 69 girls); SaR = stand-and-reach test; star run = star coordination run; participation = sports club participation; *df* = degrees of freedom; *ES* = effect size.

## Discussion

To the authors’ knowledge, this is the first study that investigated in a longitudinal approach the effects of living area and sports club participation on physical fitness development in healthy children from classes 3 to 6. The main findings can be summarized as follows: (1) over the 4-year study period, children living in urban as compared to rural areas showed a significantly better performance development for upper- (1-kg ball push) and lower-extremity muscle strength (triple hop test) and (2) children participating in sport clubs showed a significantly better performance development over the 4-year study period for measures of endurance (9-min run) and lower-extremity muscle strength (triple hop test) as compared to their non-participating peers.

### Living area and the development of physical fitness

In addition to the already existing cross-sectional studies, the present investigation provided data originating from a longitudinal study approach over a 4-year experimental period. As a result, it was found that living area has a positive effect on the development of physical fitness in children in terms of significantly better performance increments for children living in urban compared to rural areas. This was detected in significant findings for upper- (1-kg ball push) and lower-extremity muscle strength (triple hop test).

It is of interest to note that our results regarding better physical fitness in urban as compared to rural children are in contrast to most of the studies reported in the literature [11-14,23,24]. This discrepancy can most likely be explained by looking at the specific fitness items that were applied in the different studies. In fact, better values for upper- (1-kg ball push) and lower-extremity muscle strength (triple hop) were found in the present study, whereas better endurance performances (20-m shuttle run; 1-mile run) were detected in other studies [11-14]. Another reason that might account for the differences in findings, results from the methodological approaches that were used to distinguish between urban and rural living areas (e.g., number of inhabitants). In contrast to our study (urban: > 10,000 inhabitants), Tambalis et al. [25] used a cut-off value of > 5,000 inhabitants to differ between urban and rural living areas. As a consequence, areas that we defined as rural (i.e., ≤ 10,000 inhabitants) were classified as urban in the aforementioned study [25]. Additionally, in the present study the definition of rural and urban living areas was based on a federal statistics report. In contrast, Pena Reyes et al. [26] and Tsimeas et al. [27] did not report the respective reference for their classification procedure. Furthermore, the developing status of the country (i.e., industrialized country vs. emerging economy) in which the study was carried out could also have an impact. The present study was conducted in an industrialized country (i.e., Germany), whereas the study of Karkera et al. [12] investigated children from India, which is an emerging economy. In fact, it has been reported that children’s physical fitness and development differs between low- and high-income economies [11,28,29].

### Sports club participation and the development of physical fitness

The results of this study illustrated that, irrespective of living area, children participating in sports clubs showed better physical fitness development than their non-participating peers. The findings of our longitudinal approach are in accordance with that of cross-sectional studies concerning a positive association of sports club participation and physical fitness [10,15,16]. For example, Ara et al. [15] showed significantly better results in endurance (20-m shuttle run), lower-extremity muscle strength (squat jump), and speed (sprint tests over 30 and 300 m) in boys (8–11 years) participating in extracurricular sports activities as compared to their non-participating peers. In the present study, the effects of sports club participation on physical fitness development were found as a trend regarding endurance (9-min run) and as a significant finding for lower-extremity muscle strength (i.e., triple hop).

Our finding regarding better physical fitness development in sports club participating children may be attributed to the formal and structured organizational frame in which physical activity takes place. In fact, Silva et al. [30] reported for boys and girls aged 11–18 years that engagement in organized sports outside school was associated with a higher level of moderate to vigorous physical activity (MVPA). Participation in organized extracurricular sports seems to be of great public health importance considering the fact that the recommended amount of at least 60 minutes of moderate to vigorous physical activity a day [31] is not sufficiently achieved through physical education classes at school. More specifically, the average amount of time spent in physical education classes during primary school in Germany amounts to 135 minutes per week (i.e., 3 × 45 min/week). This is not enough to fulfill the above-mentioned physical activity guidelines provided by the World Health Organization [31]. Additionally, a study conducted with 6- to 12-year-old boys revealed that physical education lessons covered only 11% of the total daily MVPA [32]. In the same study, it was shown that organized youth sports covered another 23% of the total daily MVPA. Therefore, it is necessary to search for strategies inside and outside school to increase children’s physical activity and fitness levels. Organized extracurricular sports such as participation in a sports club appear to be a promising means to increase physical activity [30] and to enhance health-related behavior in youth [33]. Further, the arrangement of social commitments is particularly important during adolescence to provide additional stimuli to be physically active [34]. In this context, sports clubs may represent a natural environment adolescents like to commit themselves to because they can practice sports with their peers. For example, Carreres-Ponsoda et al. [35] observed in 12–19 year old boys and girls that those participating in out-of-school sport programs have significantly higher levels of self-efficacy, pro-social behavior as well as personal and social responsibility compared to their peers who do not participate in such programs.

In summary, due to the link between physical fitness and several health-related outcomes in youth [3,36] as well as the tracking of physical fitness from youth to adulthood [6,37], more emphasis should be laid on establishing sports clubs as an easy and attractive means to promote health-enhancing daily physical activity. However, it should be noted that additional factors (e.g., time spent watching TV or playing computer games) that were not included in this study may also have an impact on physical fitness development and should therefore be targeted in future studies.

## Conclusions

Findings from the present study indicate that the development of physical fitness is positively affected by living area and sports club participation. More specifically, children living in urban areas and children participating in sports clubs were fitter and fitness progressed faster than in their counterparts in terms of endurance (9-min run), upper- (1-kg ball push) and lower-extremity muscle strength (triple hop test). From a health perspective, these are important findings because deficits in muscle strength represent intrinsic injury and fall-related factors in youth [38,39]. Additionally, a high level of cardiorespiratory fitness is related to a lower risk in many health-related outcomes (e.g., waist circumference, blood pressure, insulin level) in children [40]. As a consequence sports club programs offering appealing arrangements could be a good means to increase physical fitness in children living in rural areas.

## Competing interests

The authors declare that they have no competing interests.

## Authors’ contributions

TM, KG, and UG analyzed the data and wrote the manuscript. DW, KG, MH, and UG contributed to the study design, data collection and critical review of draft manuscripts. TM, UG, MH, and DW assisted with the statistical analysis and interpretation of data. All the authors read and approved the final manuscript.

## Pre-publication history

The pre-publication history for this paper can be accessed here:

http://www.biomedcentral.com/1471-2458/14/499/prepub
